# Human regional tumour lymph nodes: alterations of micro-architecture and lymphocyte subpopulations.

**DOI:** 10.1038/bjc.1980.8

**Published:** 1980-01

**Authors:** O. Eremin, P. Roberts, D. Plumb, J. P. Stephens

## Abstract

Axillary lymph nodes draining mammary carcinoma showed an alteration of both micro-architectue and lymphocyte subpopulations. Lymph nodes with a normal or increased T and/or B lymphocyte compartment (assessed by histology) had a low incidence of nodal tumour spread, whilst hypocellularity of the T- or B-lymphocyte-dependent areas was associated with a significant increase in metastatic invasion. Tumour-draining lymph nodes, in particular the more proximal ones, were often enlarged, spherical and tense due to an increased cellular content, predominatly B lymphocytes and their various subsets. The increased number and percentage of B lymphocytes was associated with follicular hyperplasia and prominent germinal centres. Lymph nodes with a prominent paracortex tended to have a higher ratio of T to B lymphocytes than nodes with a hypocellular paracortical area, but in many instances both the T- and B-lymphocyte-dependent areas were increased. There was no correlation between a particular axillary-node lymphocyte subpopulation pattern (assessed by surface markers) and the size, degree of necrosis, inflammatory infiltrate or histologic type of breast carcinoma, or the presence of metastatic node invasion.


*Br. J. Cancer* (1980) **41**, 62

### HUMAN REGIONAL TUMOUR LYMPH NODES: ALTERATIONS OF MICRO-ARCHITECTURE AND LYMPHOCYTE SUBPOPULATIONS

O. EREMIN, P. ROBERTS*, D. PLUMB AND J. P. STEPHENS*

*From the Division of Immunology, Department of Pathology, University of Cambridge, and *Norfolk and Norwich Hospital, St Stephen's Road, Norwich, Norfolk*

Received 29 June 1979 Accepted 5 September 1979

**Summary.—Axillary lymph nodes draining mammary carcinoma showed an alteration of both micro-architecture and lymphocyte subpopulations. Lymph nodes with a normal or increased T and/or B lymphocyte compartment (assessed by histology) had a low incidence of nodal tumour spread, whilst hypocellularity of the T- or B-lymphocyte-dependent areas was associated with a significant increase in metastatic invasion. Tumour-draining lymph nodes, in particular the more proximal ones, were often enlarged, spherical and tense due to an increased cellular content, predominantly B lymphocytes and their various subsets. The increased number and percentage of B lymphocytes was associated with follicular hyperplasia and prominent germinal centres. Lymph nodes with a prominent paracortex tended to have a higher ratio of T to B lymphocytes than nodes with a hypocellular paracortical area, but in many instances both the T- and B-lymphocyte-dependent areas were increased. There was no correlation between a particular axillary-node lymphocyte subpopulation pattern (assessed by surface markers) and the size, degree of necrosis, inflammatory infiltrate or histologic type of breast carcinoma, or the presence of metastatic node invasion.**

ENLARGEMENT of lymph nodes adjacent to various solid tumours is a common finding in man. As early as the beginning of this century a number of gynaecologists had reported pelvic lymphadenopathy in some cases of carcinoma of the cervix (Sampson, 1906; Wartman, 1959).

Histological assessment of lymph nodes draining a variety of solid tumours (carcinoma of breast, cervix, colon, bladder, lung and head and neck) has revealed, in many instances, an alteration of nodal microarchitecture (*e.g.* Black *et al.*, 1953; Hamlin, 1968; Tsakraklides *et al.*, 1973; 1974; Patt *et al.*, 1975). This altered cellular organisation has been associated in a number of studies with a diminished incidence of tumour spread to the regional lymph nodes (*e.g.* Berg, 1956; Black & Speer, 1958; Anastassiades & Pryce, 1966; Tsakraklides *et al.*, 1973). Many authors have also reported an improved prognosis in those patients whose regional lymph nodes show an altered reactivity (*e.g.* Black *et al.*, 1953; Wartman, 1959; Hamlin, 1968; Silverberg *et al.*, 1970). Recently, alterations of lymphocyte subsets have also been described in tumour-draining lymph nodes (Richters & Kaspersky, 1975; Tsakraklides *et al.*, 1975*a*; Eremin *et al.*, 1976; Saxon & Portis, 1977).

In this paper we present further evidence substantiating such alterations in micro-architecture and lymphocyte subpopulations in lymph nodes (axillary) draining mammary carcinoma, and attempt to establish a correlation between the altered lymphoid compartments and the lymphocyte subpopulations detected in such lymph nodes. We also examine the relationship between these alterations in the lymph nodes and the prevalence of tumour metastasis.

### MATERIALS AND METHODS

### *Clinical material*

Forty patients with mammary carcinoma, clinically localised to the breast and axilla, and numerous patients with a variety of solid tumours (carcinoma of stomach, colon, rectum and lung) were studied. All patients with mammary carcinoma had a mastectomy and axillary clearance, and in 31 cases immunohistological assessment was carried out on a total of 157 axillary lymph nodes (mean of 5 per axilla). Draining lymph nodes were dissected out from the specimens in theatre, immediately after resection of the tumour. Normal lymph nodes were obtained during operations for non-malignant, non-inflammatory well-defined surgical conditions. Lymph nodes draining areas of inflammation were obtained during diagnostic or therapeutic surgical procedures.

### *Lymphocyte preparation*

Lymph nodes were cleared of fat and fascia, placed in a small pot containing tissue culture medium (TCM) and gently ballooned by injection with TCM through several puncture sites. Cells spilled out during this procedure, and the remaining lymphocytes were isolated by teasing apart the swollen node. The fibrous debris was allowed to sediment and the cell-enriched supernatant removed through a 21-gauge needle. TCM contained RPMI 1640, 100 iu penicillin/ml, 100 $\mu$g streptomycin/ml, 0·7 g sodium bicarbonate/l and 25mM Hepes buffer and 10% heat-inactivated foetal calf serum. To remove contaminating polymorphs and monocytes, the cell suspension was incubated at 37°C for 1 h with carbonyl iron and the phagocytic cells removed by a magnet.

### *Lymphocyte markers*

The various lymphocyte subpopulations were determined using lymphocyte markers, the methodology of which has been described previously (Eremin *et al.*, 1976). The non-specific sheep-RBC rosette was used to determine the T-lymphocyte population (Brown & Greaves, 1974). The B lymphocyte population (bearing surface immunoglobulin, sIg) and the various subpopulations bearing different classes of immunoglobulins (IgG, IgM, IgA, IgD and IgE) were estimated by the direct antiglobulin rosetting (DAR) reaction (Coombs *et al.*, 1977).

The antiglobulin reagent used to detect sIg was a rabbit anti-human Fab. Human IgG was pepsin treated, the digest reduced with mercaptoethanol, treated with iodoacetamide and passed through Sephadex G100. The class-specific sheep anti-human immunoglobulins were prepared at the Immunodiagnostics Research Laboratory of the Department of Experimental Pathology, Birmingham. The following preparations were used: (a) anti-IgG, produced by inoculation of pooled human Fc($\gamma$), (b) anti-IgM, produced by inoculation of a pool of 10 macroglobulin proteins, (c) anti-IgA, obtained by injection of a pool of 3 myeloma proteins, (d) anti-IgD, obtained by injection of human Fc ($\delta$) and (e) anti-IgE, prepared by inoculation of myeloma proteins. The anti-IgG serum was absorbed with insoluble Fab, the anti-IgA and anti-IgM with insoluble human cord serum, and the anti-IgD and anti-IgE with solid human whole serum. The specificities of the sheep antisera (except anti-IgE) were further characterized by reverse passive haemagglutination of indicator cells (ox red cells linked with sheep antisera) with purified immunoglobulins (Coombs *et al.*, 1978).

Initially the sIg-bearing lymphocyte population was detected by the mixed antiglobulin rosetting (MAR) reaction (Hallberg *et al.*, 1973*a*). This assay, however, has been shown to detect the same number of sIg-bearing lymphocytes as the DAR test (Coombs *et al.*, 1977) and the data from both assays have been pooled.

Fc-receptor-bearing lymphocytes were detected by opsonic adherence of ox red blood cells coated with a subagglutinating dose of rabbit IgG anti-ox-RBC antibody (Hallberg *et al.*, 1973*b*). C3-receptor-bearing lymphocytes were estimated by rosette formation with ox RBC sensitized with rabbit IgM anti-ox-RBC antibody and mouse complement (C5 deficient) (Eremin *et al.*, 1976).

### *Histological assessment*

Each lymph node was assessed by one histopathologist on two separate occasions, and the reactivity of the nodes was noted. The following features were scored as normal, increased or depleted: prominence of lymphoid follicles, presence and reactivity of germinal centres, prominence of paracortex and post-capillary venules, sinus histiocytosis and plasma-cell infiltration.

Sections of the primary breast tumour were assessed and significant lymphocyte infiltration was noted.

### RESULTS

### *Lymphocyte preparation*

The lymphnode cell-isolation technique produced 98% viable cells of which 98% were lymphocytes. The purity of the preparations was confirmed by smears stained with May-Grunwald-Giemsa and Sudan Black. The viability was assessed by phase-contrast microscopy.

### *Lymphocyte populations*

*Lymph node subpopulations.*—Some of these data have been previously published elsewhere (Eremin *et al.*, 1976). Table I shows that lymph nodes in the axilla draining mammary carcinoma show a significant alteration of lymphocyte subsets from normal lymph nodes. This altered lymphocyte pattern, however, was seen irrespective of whether there had been tumour spread to the axillary nodes. There was a substantial reduction in the percentage of the T-lymphocyte population, and a corresponding increase in the percentage of the B-lymphocyte population and its subset of Fc- and C3-receptor-bearing lymphocytes, but mainly C3-receptor-bearing lymphocytes.

Lymph nodes draining a variety of other solid tumours (carcinoma of stomach, colon, rectum and lung) showed a similar alteration of lymphocyte subsets, as did lymph nodes, in different anatomical sites, draining areas of sub-acute or chronic inflammation.

Examination of the sIg-bearing lymphocyte population revealed (Table II) that, in normal lymph nodes, IgM was the predominant class of immunoglobulin detected on the lymphocyte surface, being present on 22 ± 5% of the lymphocytes investigated. Other classes, such as IgG, IgA and IgD, were present to a lesser degree, but were fairly evenly distributed, whilst IgE was found on 1 ± 1% of lymphocytes studied. These values, in association with the data from the other lymph nodes, suggested that many B lymphocytes possessed 2 or more immunoglobulin classes on their surface membrane, and this was confirmed by mixed rosetting assays (unpublished).

Inflammatory lymph nodes, although showing an increase in the percentage of lymphocytes bearing total sIg, showed a similar pattern of class distribution, with no significant alteration of any particular Ig class. Regional tumour lymph nodes, draining a variety of solid tumours (carcinoma of stomach, colon and lung) and situated in close proximity to the tumour,

TABLE I.—*Percentage of lymphocyte subpopulations in human lymph nodes* (*mean* ± *s.d.*)

| Lymphocyte source | No. | T rosettes | Fc rosettes | C3 rosettes | sIg rosettes† |
|---|---|---|---|---|---|
| (A) Normal lymph nodes | 27 | 71 ± 6 | 23 ± 4 | 33 ± 6 | 26 ± 5 |
| (B) Axillary lymph nodes | 15 | 53 ± 12** | 36 ± 10** | 47 ± 12*** | 46 ± 8* |
| (C) Axillary lymph nodes | 12 | 55 ± 8** | 33 ± 9** | 48 ± 7*** | 45 ± 7* |
| (D) Tumour lymph nodes | 26 | 56 ± 12** | 39 ± 13** | 49 ± 13*** | 45 ± 10* |
| (E) Inflammatory lymph nodes | 15 | 52 ± 11** | 33 ± 10** | 50 ± 8*** | 46 ± 10* |

† Measurements of sIg-bearing cells made with either the MAR (early results) or the DAR (later results) assays.

(A) Similar results in nodes from different anatomical sites (Eremin *et al.*, 1976).

(B) Tumour localised to the breast.

(C) Tumour spread to the axilla, but only tumour-free nodes used.

(D) Regional lymph nodes draining a variety of solid tumours (carcinoma of the stomach, colon, rectum and lung). Only tumour-free nodes.

(E) Draining areas of chronic inflammation.

Statistical significance of the differences in the various lymphocyte subpopulations was assessed by a one-way analysis of variance.

No statistically significant differences between B and C in the various lymphocyte subpopulations determined.

Asterisks indicate significant differences from (A): * = $P < 0 \cdot 05$; ** = $P < 0 \cdot 001$; *** = $P < 0 \cdot 0001$.

TABLE II.—*Percentage of various immunoglobulin classes on human lymphnode lymphocytes (mean ± s.d.)*

| Lymphocyte source | No. | sIg | IgG | IgM | IgA | IgD | IgE |
|---|---|---|---|---|---|---|---|
| (A) Normal lymph nodes | 8 | 29 ± 4 | 17 ± 9 | 22 ± 5 | 16 ± 4 | 13 ± 5 | 1 ± 1 |
| (B) Axillary lymph nodes | 6 | 50 ± 7** | 28 ± 7* | 34 ± 12* | 31 ± 8* | 23 ± 6 | 3 ± 3 |
| (C) Axillary lymph nodes | 6 | 49 ± 11** | 27 ± 6* | 33 ± 8* | 20 ± 8 | 31 ± 10* | 3 ± 3 |
| (D) Tumour lymph nodes | 7 | 58 ± 10** | 43 ± 13* | 48 ± 11* | 40 ± 6* | 43 ± 15* | 4 ± 3 |
| (E) Inflammatory lymph nodes | 7 | 43 ± 7** | 22 ± 9 | 26 ± 12 | 17 ± 7 | 21 ± 9 | 3 ± 2 |

The Direct Antiglobulin Rosetting assay (DAR) was used to measure sIg and the various Ig classes.
A–E as in Table I; C shows shows reversal of IgA:IgD ratio.
Statistical significance of the differences in the various Ig classes was assessed by a one-way analysis of variance.
Statistically significant differences from A are shown by *($P < 0{\cdot}05$) and ** ($P < 0{\cdot}005$).
Comparison of (B) with (C) shows no statistically significant differences in the distribution of Ig classes, apart from % of IgD and IgA.

showed a marked increase in the percentage of cells bearing all the Ig classes. In contrast to the findings in normal and inflammatory lymph nodes, however, excluding IgE, there was no predominance of any single Ig class on the lymphocyte surface.

Table II also shows the Ig class spectrum of axillary lymph nodes draining mammary carcinomas. As with the other tumour and inflammatory lymph nodes, the percentage of lymphocytes bearing IgG and IgM was substantially raised. Metastatic tumour spread to the axilla, however, modified the IgA- and IgD-bearing lymphocyte subpopulations. The IgA-bearing population was only slightly increased while the IgD was quite prominent. This pattern was reversed in tumour-free axillae. A larger sampling batch is required to confirm this difference unequivocally.

*Proximity of lymph node to tumour.*—

TABLE III.—*Relationship of tumour lymphnode lymphocyte subpopulations and the proximity of the node to the tumour**

| Tumour type | Proximity of lymph node to the tumour | Mean % lymphocyte subpopulations in tumour lymph nodes | | | |
|---|---|---|---|---|---|
| | | T rosettes | Fc rosettes | C3 rosettes | sIg rosettes† |
| Ca colon‡ | Proximal | 38 | 68 | 68 | 65 |
| | Proximal | 33 | 75 | 77 | 73 |
| Ca of colon§ | Proximal | 36 | 40 | 70 | 66 |
| | Proximal | 44 | 25 | 59 | 50 |
| Ca caecum‖ | Proximal | 48 | 55 | 57 | 55 |
| | Distal | 68 | 34 | 37 | 34 |
| Ca stomach¶ | Proximal | 57 | 40 | 55 | 50 |
| | Distal | 71 | 30 | 40 | 31 |
| Ca lung** | Proximal | 40 | 43 | 59 | 57 |
| | Distal | 55 | 50 | 52 | 42 |
| Ca lung†† | Proximal | 68 | 21 | 32 | 26 |
| | Distal | 70 | 33 | 32 | 25 |

* Proximal refers to primary draining lymph nodes and distal to secondary, tertiary etc. draining lymph nodes.
† Ascertained by the Direct Antiglobulin Rosetting assay (DAR).
‡, § Adjacent pairs of paracolic lymph nodes from 2 Ca colon specimens.
‖ Ileocaecal lymph node close to ileocaecal junction (proximal) and mesenteric lymph node close to root of mesentery (distal).
¶ Subpyloric lymph node (proximal) and cardial lymph node (distal) from carcinoma of the gastric antrum.
**, †† Hilar lymph nodes (proximal) and paratracheal lymph nodes (distal) from 2 bronchogenic tumours. In the former (7), alteration of lymphocyte subsets found in both the proximal and distal draining lymph nodes. In the latter (8), no alteration of lymphocyte subsets detected in either the proximal or distal draining lymph nodes.

The position of the lymph node in relation to the growing solid tumour may markedly alter the lymphocyte subpopulations of that lymph node (Table III). This is less well defined with axillary lymph nodes draining mammary carcinoma due to the confined space of the axilla. The lymphatic field of drainage of gastro-intestinal or bronchogenic tumours is much more extensive and more readily defined into proximal (primary draining) and distal (secondary or tertiary draining) lymph nodes.

Lymph nodes in close proximity to the tumour (*e.g.*, carcinoma of colon) often showed a profound alteration (inversion) of the T:B lymphocyte ratio. More distal (secondary or tertiary draining) lymph nodes may either fail to show an alteration of this ratio (*e.g.*, carcinoma of caecum and stomach) or show a similar subpopulation spectrum to that found in the more proximal lymph node (*e.g.*, carcinoma of lung). At times both the proximal and distal lymph nodes had a normal lymphocyte subpopulation pattern (*e.g.*, carcinoma of lung).

*Physical characteristics of lymph nodes.*—Certain physical characteristics of the draining lymph nodes may serve as a guide to the nodal reactivity and consequent alteration of lymphocyte subpopulations within that node. Size, although an obvious parameter, could by itself by misleading, as shown by the data in Table IV. Some large nodes (2 cm) had a normal pattern of lymphocyte subpopulations, whereas many small nodes (0·5 cm) showed a pronounced alteration of this pattern.

The shape and consistency of the nodes were very important physical parameters. Oval or reniform, flattened or thin and soft nodes, even when large, had a normal subpopulation pattern. On the other hand, spherical or rounded, tense and oedematous or rubbery and firm lymph nodes, even when small, showed a marked alteration of their lymphocyte subpopulations. The change in shape and consistency was a reflection of the pronounced increase in the total cellular content of the node. Even these parameters, however, were sometimes misleading, as some normal but large lymph nodes in the axilla were swollen by a fatty central core, and felt firm.

### *Lymphnode histology*

Axillary lymph nodes, removed from mastectomy specimens, showed, on histological assessment, evidence of alteration of their micro-architecture. In most cases the histological findings for any given set of lymph nodes from a particular axilla were uniform, and the individual lymphnode data pooled. Where, on occasion, there was variation amongst the lymph nodes of any one axilla the predominant

TABLE IV.—*Relationship of tumour lymphnode lymphocyte subpopulations to physical characteristics of the node**

| Lymph node size—widest diameter (cm) | Lymphnode shape and consistency | Percentage lymphocyte subpopulations in axillary lymph nodes† | | | |
|---|---|---|---|---|---|
| | | T rosettes | Fc rosettes | C3 rosettes | sIg rosettes‡ |
| 2·0 | Oval, firm, swollen by a fatty core§ | 75 | 10 | 21 | 27 |
| 2·0 | Reniform, thin, soft | 78 | 9 | 22 | 20 |
| 2·0 | Spherical, rubbery, hard | 33 | 29 | 63 | 65 |
| 0·5 | Spherical, tense, oedematous, firm | 50 | 30 | 55 | 50 |
| 0·5 | Rounded, tense, hard | 45 | 36 | 51 | 50 |
| 0·5 | Oval, thin, soft | 70 | 25 | 32 | 29 |

* Results for 6 individually selected lymph nodes.
† Regional draining nodes from the tumour-free axillary contents of the mastectomy specimens.
‡ By DAR, assay.
§ Some normal but large lymph nodes in the axilla are swollen by a fatty central core, and feel firm. These can be mistaken for hyperplastic, reactive lymph nodes at operation.

TABLE V.—*Micro-architecture of axillary lymph nodes and the incidence of tumour spread*

| Incidence of lymphnode invasion by tumour* | % | Histological parameters: Type | Grade† | Incidence in 31 axillae | % |
|---|---|---|---|---|---|
| 13/80 | 16 | Paracortex | Prominent | 16 | 52 |
| 2/48 | 4 | | Normal | 10 | 32 |
| 12/28 | 47 | | Depleted | 5 | 16 |
| 12/90 | 13 | Sinus histiocytosis | Prominent | 16 | 52 |
| 2/43 | 4 | | Normal | 8 | 25 |
| 14/31 | 45 | | Depleted | 7 | 23 |
| 22/129 | 17 | Lymphoid follicles | Prominent | 22 | 70 |
| 2/22 | 9 | | Normal | 6 | 20 |
| 5/16 | 32 | | Depleted | 3 | 10 |
| 10/81 | 12 | Germinal centres | Prominent | 10 | 32 |
| 6/44 | 14 | | Normal | 16 | 52 |
| 12/27 | 45 | | Depleted | 5 | 16 |

* Expressed as the number of invaded lymph nodes (histologically proven) over the total lymph nodes examined.

† For the sake of simplicity, + to + + + was scored as prominent, and − to − − − as depleted.

alteration was selected. These findings, on 157 lymph nodes in 31 axillae, are summarised in Table V as individual histological parameters, but in most lymph nodes more than one parameter was altered.

*Paracortex.*—The paracortical area, which represents the thymus-dependent lymphoid compartment of the lymph node, showed wide histological fluctuations, but the various histological patterns, in general, paralleled the T-lymphocyte populations (%) (Table VI). That the T-lymphocyte percentages were not higher in lymph nodes with normal or prominent cortical areas was because most nodes showed evidence of increases of both the the T- and B-lymphocyte-dependent areas.

In one-third of the examined axillae, the lymph nodes had a normal paracortical area and a very low incidence (4%) of secondary tumour spread. In half of the axillae there was an increase in the paracortical area of the lymph nodes

TABLE VI.—*Micro-architecture of axillary lymph nodes and their lymphocyte subpopulations*

| Histological parameters: Type | Grade*: Prominent | Normal | Depleted | Lymphocyte subpopulations |
|---|---|---|---|---|
| Sinus histiocytosis | 54† | 56 | 54 | T rosettes (%) |
| Paracortex | 60 | 54 | 49 | T rosettes (%) |
| Lymphoid follicles | 34 | 28 | 29 | Fc rosettes (%) |
| | 46 | 40 | 36 | ‡C3 rosettes (%) |
| | 45 | 41 | 34 | Ig rosettes (%) |
| Germinal centres | 35 | 30 | 27 | Fc rosettes (%) |
| | 52 | 45 | 40 | ‡C3 rosettes (%) |
| | 48 | 42 | 35 | Ig rosettes (%) |

*For the sake of simplicity, + to + + + was scored as prominent and − to − − − as depleted.

†Values for each lymphocyte subpopulation are expressed as the mean of all the values found in the various lymph nodes examined, showing the indicated grade of histological parameter.

‡There is a close correlation between the C3-receptor-bearing and sIg-bearing lymphocyte populations, and either can be regarded as representative of the B-lymphocyte subpopulation, although the percentage of the former tends to be slightly higher.

and a low incidence (16%) of secondary tumour spread. In a small number of axillae, the lymph nodes had markedly reduced paracortical areas and a high incidence of tumour invasion (47%).

*Sinus histiocytosis.*—Changes in sinus histiocytosis paralleled those of the paracortical compartment in only about two-thirds of the cases and the T-lymphocyte percentages were unaltered by histological fluctuations (Table VI).

In half the axillae examined, the lymph nodes showed prominent sinus histiocytosis and a low incidence of metastatic tumour spread (13%). One-quarter of the axillae had a normal pattern and a very low incidence of tumour spread (4%). Depletion of sinus histiocytes was detected in the remaining quarter of the lymph nodes, which had a high incidence of tumour metastasis.

*Lymphoid follicles.*—The Fc-receptor, C3-receptor and sIg-bearing lymphocyte subpopulations were increased in lymph nodes with prominent lymphoid follicles (Table VI), which represent the B-lymphocyte-dependent compartment of the node. The sIg-bearing population showed a close correlation with the C3-receptor-bearing subpopulation.

Table V shows that, in one-fifth of the axillae examined, the lymphoid follicular area was normal and the incidence of tumour spread low (9%). In 70% of the axillae, there was an increase in the lymphoid follicles of the nodes. Although the percentage of node involvement was increased to 17%, it was still less than that in the remaining small number of axillae (10%) where the follicular area was diminished and 32% of the nodes were invaded by tumour.

*Germinal centres.*—Germinal centres were prominent in some of the lymph nodes with an increased number of lymphoid follicles (Table VI). The Fc-receptor-bearing and sIg-bearing populations were the same in these nodes as in those with prominent follicles but normal germinal-centre architecture. Lymph nodes with prominent germinal centres, however, tended to have a higher percentage of C3-receptor-bearing lymphocytes.

Half of the axillae examined had a normal germinal-centre architecture with a low incidence of tumour spread (14%) (Table V). One third of cases had increased numbers of germinal centres and a persistent low incidence of metastatic invasion (12%). In a small number of axillae, the lymph nodes had a diminished number of germinal centres and a prominent increase in the percentage of tumour-involved lymph nodes (45%).

*Medulla plasma cells:* Most of the lymph nodes (60%) had a normal number of plasma cells in the medulla. The percentage of sIg-bearing lymphocytes in the nodes showed no correlation with the histological assessment of plasma cells.

Similarly no obvious correlation was detected between the number of plasma cells in the medulla and the incidence of tumour invasion of the axilla (data not shown).

*Post-capillary venule.*—The specialized post-capillary venule (high-walled endothelium) in the paracortical area with its surrounding cuff of lymphocytes, was assessed histologically in 24 lymph nodes. Prominence of, and an increase in, post-capillary venule numbers and cellularity of the surrounding lymphocyte cuff was found to parallel paracortical hyperplasia, and probably represented an increased lymphocyte migration into the paracortical area (Goldschneider & McGregor, 1968). This increased migration may or may not be selective for a particular lymphocyte subpopulation.

*Tumour histology*

Thirty-one mammary carcinomas, varying in size from 1 to 5 cm, were assessed histologically. Twenty-five were found to be intraductal and invasive, 1 was medullary, 2 were lobular and invasive and 3 were poorly differentiated and invasive. Within each tumour there was a wide variation in the degree of neoplasia, and it was not possible, in the samples available, to grade them.

A prominent lymphocytic inflammatory response, primarily at the tumour edge, was found in 7 specimens. In those cases where no axillary invasion by tumour had occurred, 6/19 (32%) had evidence of a prominent cellular infiltration. In those cases, on the other hand, where tumour had spread to the axillary nodes, 1/15 (7%) had evidence of a prominent cellular infiltration. Hence a high percentage (86%) of tumours with a lymphocytic infiltration had no evidence of tumour spread to the axillary lymph nodes.

We were unable to show, in the samples studied, any obvious correlation between the axillary lymphnode lymphocyte subsets and the size of the tumour (1–5 cm) the degree of tumour necrosis (+ to + + +) the histological type of tumour or the presence or absence of a lymphocytic infiltration at the tumour edge.

### DISCUSSION

Evidence has accumulated from a number of studies in man, showing that regional tumour lymph nodes, draining a variety of solid tumours of different pathological types, show an alteration of their micro-architecture (Black *et al.*, 1953; Hamlin, 1968; Tsakraklides *et al.*, 1973; 1974; Patt *et al.*, 1975). The results of this present investigation of axillary lymph nodes draining mammary carcinoma confirms these findings. In half of the axillae examined the lymph nodes had, on histological evaluation, an increased paracortical area. Increased sinus histiocytosis was similarly seen in half of the lymph nodes. The presence of both, however, was only seen in 60% of the examined nodes. Seventy per cent of lymph nodes showed evidence of lymphoid follicular hyperplasia, whilst less than half of these had increased numbers of germinal centres. In many lymph nodes there was a concomitant increase in both the T and B lymphocyte-dependent compartments.

Histological studies of normal lymph nodes, obtained at autopsy or surgery, reveal that such prominent changes in lymphnode microarchitecture are rare in man (Black *et al.*, 1953; Tsakraklides, 1975*b*). Such changes, on the other hand, have been well documented in animals in regional lymph nodes draining skin homografts (Scothorne & McGregor, 1955; Turk, 1967) or a variety of tumours, whether spontaneous (Edwards *et al.*, 1971; Fisher, 1977) or chemically-induced (Kruger, 1967; Alexander *et al.*, 1969; Flannery *et al.*, 1975; Nelson & Kearney, 1976; Robinson *et al.*, 1977) These changes of cellular organization within a draining lymph node suggest an altered reactivity and a possible defence mechanism on the part of the host.

The present study shows that axillary lymph nodes depleted of either the T (paracortex) or B (follicles, germinal centres) lymphocyte-dependent compartments had a high incidence, in some cases up to 50%, of metastatic tumour invasion. Conversely, a normal or prominent T- or B-lymphocyte-dependent compartment was associated with a low incidence of tumour metastasis.

Regional lymph nodes with prominent germinal centres and follicular hyperplasia, draining various solid tumours (carcinoma of cervix, head and neck, and lung) have been shown by several workers to be associated with a decreased incidence of tumour spread (Tsakraklides *et al.*, 1973; Berlinger *et al.*, 1976; Di Paola *et al.*, 1977; Van Nagell *et al.*, 1977). These same authors similarly reported a low incidence of nodal invasion where there was a prominent paracortical area in the regional tumour nodes. Prominent sinus histiocytosis in axillary tumour lymph nodes has been described by a few workers as not influencing nodal metastases (Dike & Lane, 1963; Kister *et al.*, 1969). Most authors, however, describe the presence of prominent sinus histiocytosis in nodes draining carcinoma of the breast as being associated with a reduced incidence of tumour spread to such nodes (Berg, 1956; Black & Speer, 1958; Wartman, 1959; Fisher *et al.*, 1975). In the present study, lymph nodes showing diminished sinus histiocytosis had a significantly higher incidence of tumour invasion.

Axillary lymph nodes removed from the mastectomy specimens in the present study, as previously reported (Eremin *et al.*, 1976) showed a profound alteration in lymphocyte subpopulations. The percentage of T lymphocytes was reduced and there was a corresponding increase in the percentage of B lymphocytes. Both the Fc-receptor- and C3-receptor-bearing lymphocyte subpopulations were also increased, but with a persistence of the normal C3-receptor-bearing lymphocyte predominance. Tumour invasion of the axilla, however, had no effect on the lymphnode subsets detected (tumour-free nodes) and accords with the findings of Tsakraklides *et al.* (1975*a*). It was not possible, with the specimens available, to find any correlation between the histological type of tumour or its grade of malignancy, and the particular lymphocyte pattern detected. There did not appear to be any obvious correlation between the size of the growth, degree of tumour necrosis, or the presence or absence of a lymphocytic infiltrate at the periphery of the tumour and the lymphocyte subpopulations detected in the tumour-draining node.

Regional lymph nodes draining various solid tumours (carcinoma of stomach, colon and lung) showed similar alterations of lymphocyte subpopulations. These changes in the T- and B-lymphocyte subpopulations were first evident, most pronounced and often localized in the proximal draining lymph nodes, which undergo alterations of size, shape and consistency due to an increase in the total lymphocyte content. Palpation of the axilla during physical examination is often an unreliable method of assessing tumour spread (Fisher *et al.*, 1975). Similarly, the evaluation of lymphnode reactivity at operation can also be unreliable, as size alone is only a rough guide and the assessment of lymphnode shape and consistency can be difficult when the nodes are surrounded by and embedded in fat.

Lymph nodes subjected to repeated and persistent antigenic stimulation (*e.g.* draining areas of chronic inflammation) showed a pattern of T and B lymphocyte alteration similar to that found in the tumour-draining nodes. Rodent lymph nodes, stimulated by a variety of antigens, show similar changes in the T and B lymphocyte subpopulations (Onoe, 1976; Gery *et al.*, 1977). These findings suggest a comparable host defence mechanism by the human regional tumour lymph nodes.

In this study the incidence and distribution of Ig classes on the B lymphocytes was also investigated. Apart from an increase in the percentage of sIg-bearing cells, the percentage of B lymphocytes carrying the various Ig classes (IgG, IgM, IgA, IgD and IgE) was also increased in the regional tumour lymph nodes. The IgM-bearing lymphocyte subpopulation was predominant in both the normal and tumour-draining lymph nodes. This disagrees with Burtin *et al.* (1969) who found IgA to be predominant in tumour-draining nodes. Again, tumour spread to the axilla did not increase the percentage of the IgM-bearing lymphocyte subpopulations in our study, and contrasts with the findings of Richters & Kaspersky (1975). There was, however, a reversal of the IgA:IgD ratio in tumour-free axillary lymph nodes where axillary spread of tumour had occurred. A similar increase in IgD has been described by Fontaine *et al.* (1974) in lymph nodes invaded by tumour. The significance of these findings is uncertain. Lymph nodes in close proximity to tumours showed a pronounced increase in all the Ig classes, and these findings suggest that such lymphocytes (as well as others) carry at least 2 Ig classes on their cell membrane (unpublished findings; Fontaine *et al.*, 1974; Dhaliwal *et al.*, 1978).

In general, there was an association between the micro-architecture of the tumour lymph node and the lymphocyte profile of the node. Increased paracortical areas tended to have higher percentages of T lymphocytes whilst a node with a depleted paracortex tended to have a lower percentage of T lymphocytes. Similarly, follicular hyperplasia and increased numbers of germinal centres were found in nodes with increased C3-receptor- and sIg-bearing lymphocytes. Tsakraklides *et al.* (1975*a*) had reported similar findings in axillary nodes draining carcinoma of the breast.

The majority of the nodes had follicular hyperplasia, but only half had paracortical enlargement, and this was reflected in the altered T- and B-lymphocyte ratio detected in such nodes. Many lymph nodes had an increase in both the T and B lymphocyte-dependent compartments of the node, the resultant T lymphocyte percentages being therefore a sum of both the T- and B-lymphocyte population increases, and consequently reduced. In some lymph nodes a hypocellular paracortical area coexisted with a normal or prominent follicular area. Lymphocyte subpopulation estimates carried out on lymphocytes isolated from nodes showing micro-architectural heterogeneity were often comparable. This explains the lack of correlation between a particular lymphocyte-subset spectrum and nodal tumour invasion.

Most nodes showing alterations of lymphocyte subsets (and shape and size) had an increased total lymphocyte content. This is probably partly due to an increased influx of lymphocytes across the specialized post-capillary venules in the paracortical area, as evidenced in our study by the prominence and increased numbers of these venules with their surrounding lymphocytic cuffs (Parrott, 1967; Goldschneider & McGregor, 1968). Both T and B lymphocytes migrate across the specialized post-capillary venule (Nienwenhuis & Ford, 1976) and there is no evidence of selective subpopulation migration. The increased cellularity of the paracortical area probably represents a true increase in the T-lymphocyte subpopulation, but also reflects the total nodal lymphocyte increase, including the B-lymphocyte subpopulation, which following its entry across the post-capillary venule into the paracortex, remains for a few hours before migrating to the follicular area (Nienwenhuis & Ford, 1976). Some of the increased cellularity (T and B lymphocytes) of the draining tumour lymph node could be a reflection of a local immune response against the mammary tumour. It is not possible to say with certainty whether this altered nodal reactivity (local immune response) is breast-tumour specific. There was, however, no obvious correlation between any of the factors discussed above and the presence of lymphoblasts or the plasma-cell content in the medulla.

We gratefully acknowledge the help given by Professor R. R. A. Coombs in the various DAR assays and Mr R. Hanka with the statistical analysis. This study was supported by the Cancer Research Campaign.

### REFERENCES

ALEXANDER, P., BENSTED, J., DELORME, E. J., HALL, J. G. & HODGETT, J. (1969) The cellular immune response to primary sarcomata in rats. II. Abnormal responses of nodes draining the tumour. *Proc. R. Soc. Lond. Biol.*, **174**, 232.

ANASTASSIADES, O. T. & PRYCE, D. M. (1966) Immunological significance of the morphological changes in lymph nodes draining breast cancer. *Br. J. Cancer*, **20**, 239.

BERG, J. W. (1956) Sinus histiocytosis: A fallacious measure of host resistance to cancer. *Cancer*, **9**, 935.

BERLINGER, N. T., TSAKRAKLIDES, V., POLLAK, K., ADAMS, G. L., YANG, M. & GOOD, R. A. (1976) Immunologic assessment of regional lymph node histology in relation to survival in head and neck carcinoma. *Cancer*, **37**, 697.

BLACK, M. M. & SPEER, F. D. (1958) Sinus histiocytosis of lymph nodes in cancer. *Surg. Gynaecol. Obst.*, **106**, 163.

BLACK, M. M., KERPE, S. & SPEER, F. D. (1953) Lymph node structure in patients with cancer of the breast. *Am. J. Pathol.*, **29**, 505.

BROWN, G. & GREAVES, M. (1974) Cell surface markers for human T and B lymphocytes. *Eur. J. Immunol.*, **4**, 302.

BURTIN, P., LOISILLIER, F., BUFFE, D., GUILLERM, M. & GLUCKMAN, E. (1969) Immunoglobulin-producing cells in human pericancerous lymph nodes. *Cancer*, **23**, 80.

COOMBS, R. R. A., WILSON, A. B., EREMIN, O., & 5 others (1977) Comparison of the direct antiglobulin rosetting reaction with the mixed antiglobulin rosetting reaction for the detection of immunoglobulin on lymphocytes. *J. Immunol. Methods*, **18**, 45.

COOMBS, R. R. A., EDEBO, L., FEINSTEIN, A. & GURNER, B. W. (1978) The class of antibodies sensitizing bacteria measured by mixed reverse passive antiglobulin haemagglutination (MRPAH), *Immunology*, **34**, 1037.

DHALIWAL, H. S., LING, N. R., BISHOP, S. & CHAPEL, H. (1978) Expression of Immunoglobulin G on blood lymphocytes in chronic lymphocytic leukemia. *Clin. Exp. Immunol.*, **31**, 226.

DI PAOLA, M., BERTOLOTTE, A., COLIZZA, S. & COLI, M. (1977) Histology of bronchial carcinoma and regional lymph nodes as putative immune responses of the host to the tumour. *J. Thorac. Cardiovasc. Surg.*, **73**( 531.

DIKE, J. J. & LANE, N. (1963) The relation of sinus histiocytosis in axillary lymph nodes to surgical curability of carcinoma of the breast. *Am. J. Clin. Pathol.*, **40**, 508.

EDWARDS, A. J., SUMNER, M. R., ROWLAND, G. F. & HURD, C. M. (1971) Changes in the lymphoreticular tissues during growth of a murine adenocarcinoma. I. Histology and weight of lymph nodes, spleen and thymus. *J. Natl Cancer Inst.*, **47**, 301.

EREMIN, O., PLUMB, D. & COOMBS, R. R. A. (1976) T & B lymphocyte populations in human normal lymph node, regional tumour lymph node and inflammatory lymph node. *Int. Arch. Allergy Appl. Immunol.*, **52**, 277.

FISHER, E. R., GREGORIO, R. M., FISHER, B., REDMOND, C., VELLIOS, F. & SOMMERS, S. C. (1975) The pathology of invasive breast cancer. *Cancer*, **36**, 1.

FISHER, E. R. (1977) The regional lymph node in cancer: Relationship of nodal histologic findings to cytotoxicity and immunity. *Arch. Pathol.*, **101**, 152.

FLANNERY, G. R., MULLER, H. K. & NAIRN, R. C. (1975) Lymphoreticular response to a syngeneic rat tumour: Gravimetric and histological studies. *Br. J. Cancer*, **31**, 614.

FONTAINE, M., MERCIER, P. & MONGIN, M. (1974) Report of IgA, IgD, IgG and IgM secreting plasma cells in cancerous lymph nodes. *Biomedicine*, **21**, 168.

GERY, I., NAVOK, T. & STUPP, Y. (1977) Selective accumulation of cells with "B" properties in stimulated lymph nodes. *Immunology*, **33**, 727.

GOLDSCHNEIDER, I. & MCGREGOR, D. D. (1968) Migration of lymphocytes and thymocytes in the rat. I. The route of migration from blood to spleen and lymph nodes. *J. Exp. Med.*, **127**, 155.

HALLBERG, T., HAEGERT, D. G., CLEIN, G. P., COOMBS, R. R. A., FEINSTEIN, A. & GURNER, B. W. (1973*a*) Observations on the mixed antiglobulin reaction as a test for immunoglobulin bearing lymphocytes in normal persons and in patients with chronic lymphatic leukaemia. *J. Immunol. Methods*, **4**, 317.

HALLBERG, T., GURNER, B. W. & COOMBS, R. R. A. (1973*b*) Opsonic adherence of sensitized ox red blood cells to human lymphocytes as measured by rosette formation. *Int. Arch. Allergy Appl. Immunol.*, **44**, 500.

HAMLIN, I. M. E. (1968) Possible host resistance in carcinoma of the breast: A histological study. *Br. J. Cancer*, **22**, 383.

KISTER, S. J., SOMINERO, S. G., HAAGENSEN, C. D., FRIEDELL, G. H., COLLEY, E. & VARINA, A. (1969) Nuclear grade and sinus histiocytosis in cancer of the breast. *Cancer*, **23**, 570.

KRUGER, C. (1967) Morphologic studies of lymphoid tissues during the growth of an isotransplanted mouse tumour. *J. Natl Cancer Inst.*, **39**, 11.

NELSON, D. & KEARNEY, R. (1976) Macrophages and lymphoid tissues in mice with concomitant tumour immunity. *Br. J. Cancer*, **34**, 221.

NIENWENHUIS, P. & FORD, W. L. (1976) Comparative migration of B and T lymphocytes in the rat spleen and lymph node. *Cell. Immunol.*, **23**, 254.

ONOE, K. (1976) Changes in histology of the regional lymph nodes and in the proportion of T and B cell populations by oxazolone painting or LPS injection in guinea pigs. *Acta Pathol. Jpn*, **26**, 671.

PARROTT, D. M. V. (1967) The response of draining lymph nodes to immunological stimulation in intact and thymectomised animals. *J. Clin. Pathol.*, **20**, 456.

PATT, D. J., BRYNES, R. K., VARDIMAN, J. W. & COPPLESON, L. W. (1975) Mesocolic lymph node histology is an important prognostic indicator for patients with carcinoma of the sigmoid colon: An immunopathologic study. *Cancer*, **35**, 1388.

RICHTERS, S. & KASPERSKY, C. L. (1975) Surface immunoglobulin positive lymphocytes in human breast cancer tissue and homolateral axillary lymph nodes, *Cancer*, **35**, 129.

ROBINSON, G., JONES, J. A. & REES, R. C. (1977) Histological and immunological responses in the draining lymph nodes during tumour growth in rats. *Br. J. Cancer*, **36**, 430.

SAMPSON, J. A. (1906) A careful study of the parametrium in twenty-seven cases of carcinoma Cervicis Uteri and its clinical significance. *Am. J. Obstet. Gynecol.*, **54**, 433.

SAXON, S. & PORTIS, J. (1977) Lymphoid subpopulation changes in regional lymph nodes in squamous head and neck cancer. *Cancer Res.*, **37**, 1154.

SCOTHORNE, R. J. & MCGREGOR, I. A. (1955) Cellular changes in lymph nodes and spleen following skin homografting in the rabbit. *J. Anat.*, **89**, 283.

SILVERBERG, S. G., CHITALE, A. R., HURD, A. D., FRAZIER, A. B. & LEVITT, S. H. (1970) Sinus histiocytosis and mammary carcinoma, a study of 363 radical mastectomies, and a historical review. *Cancer*, **20**, 1177.

TSAKRAKLIDES, V., ANASTASSIADES, O. T. & KERSEY, J. H. (1973) Prognostic significance of regional lymph node histology in uterine cervical cancer. *Cancer*, **31**, 860.

TSAKRAKLIDES, V., OLSON, P., KERSEY, J. H. & GOOD, R. A. (1974) Prognostic significance of the regional lymph node histology in cancer of the breast. *Cancer*, **34**, 1259.

TSAKRAKLIDES, E., TSAKRAKLIDES, V., ASHIKARI, H., ROSEN, P. P., SEGAL, F. P., ROBBINS, G. F. & GOOD, R. A. (1975*a*) *In vitro* studies of axillary lymph node cells in patients with breast cancer. *J. Natl Cancer Inst.*, **54**, 549.

TSAKRAKLIDES, V., TSAKRAKLIDES, E. & GOOD, R. A. (1975*b*) An autopsy study of human axillary lymph node histology. *Am. J. Pathol.*, **78**, 7.

TURK, J. L. (1967) Cytology of the induction of hypersensitivity. *Br. Med. Bull.*, **23**, 3.

VAN NAGELL, J. R., DONALDSON, E. S., PARKER, J. C., VAN DYKE, A. H. & WOOD, E. G. (1977) The prognostic significance of pelvic lymph node morphology in carcinoma of the uterine cervix. *Cancer*, **39**, 2624.

WARTMAN, W. B. (1959) Sinus cell hyperplasia of lymph nodes regional to adenocarcinoma of the breast and colon. *Br. J. Cancer*, **13**, 189.


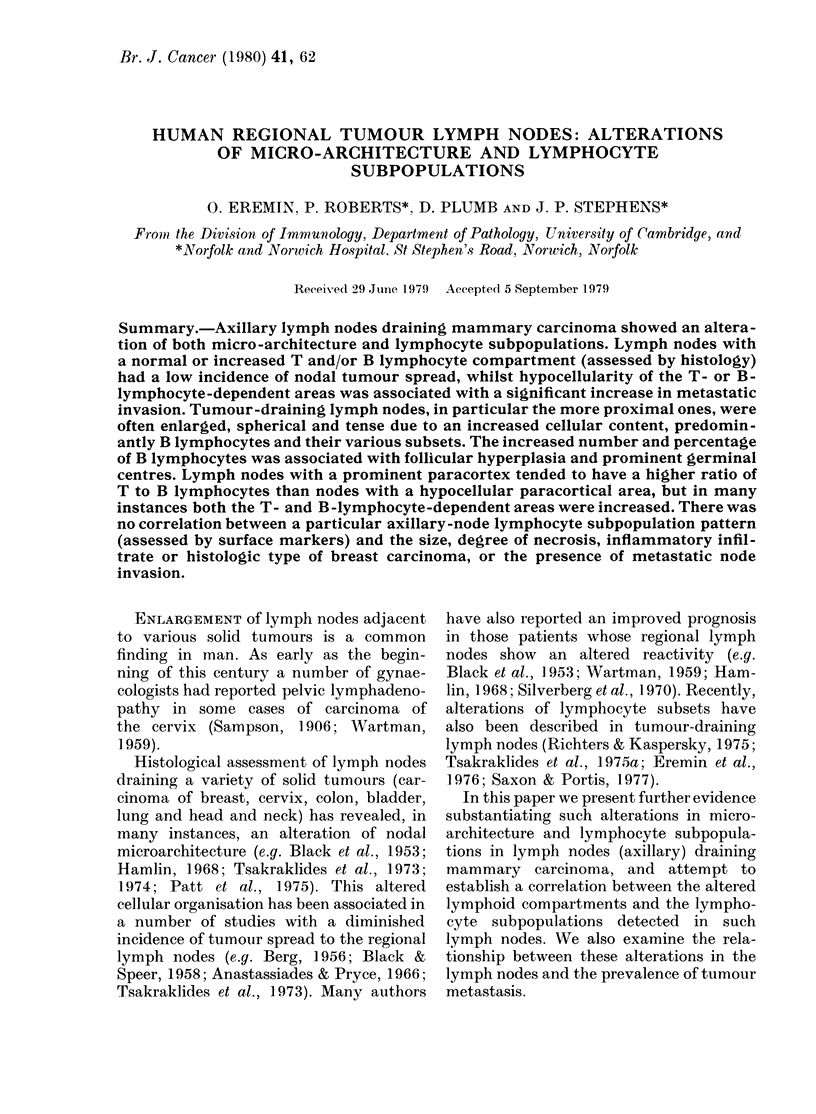

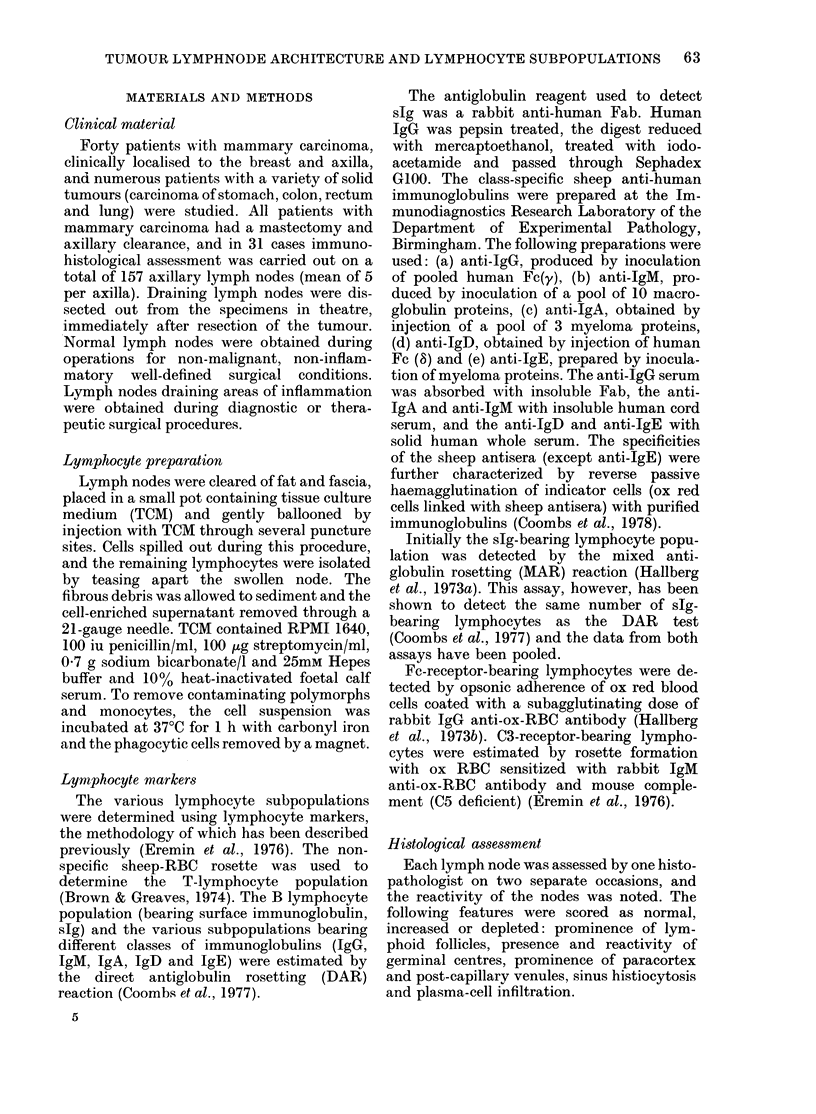

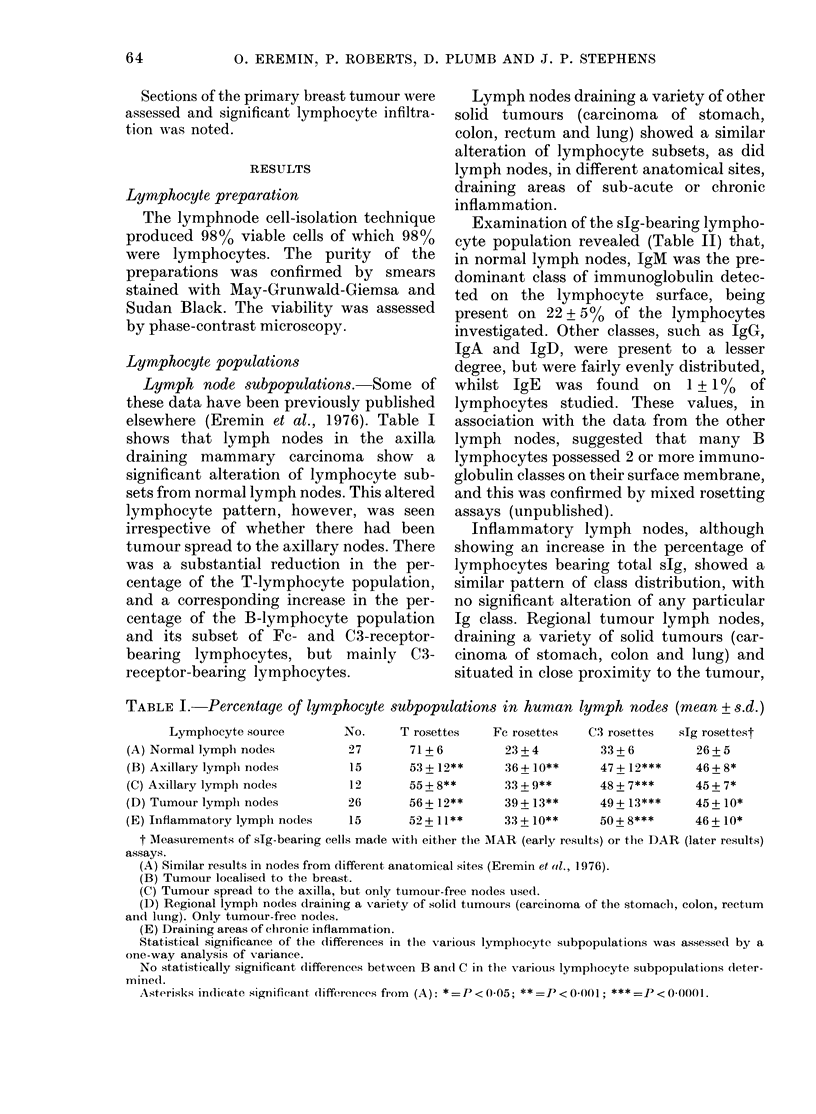

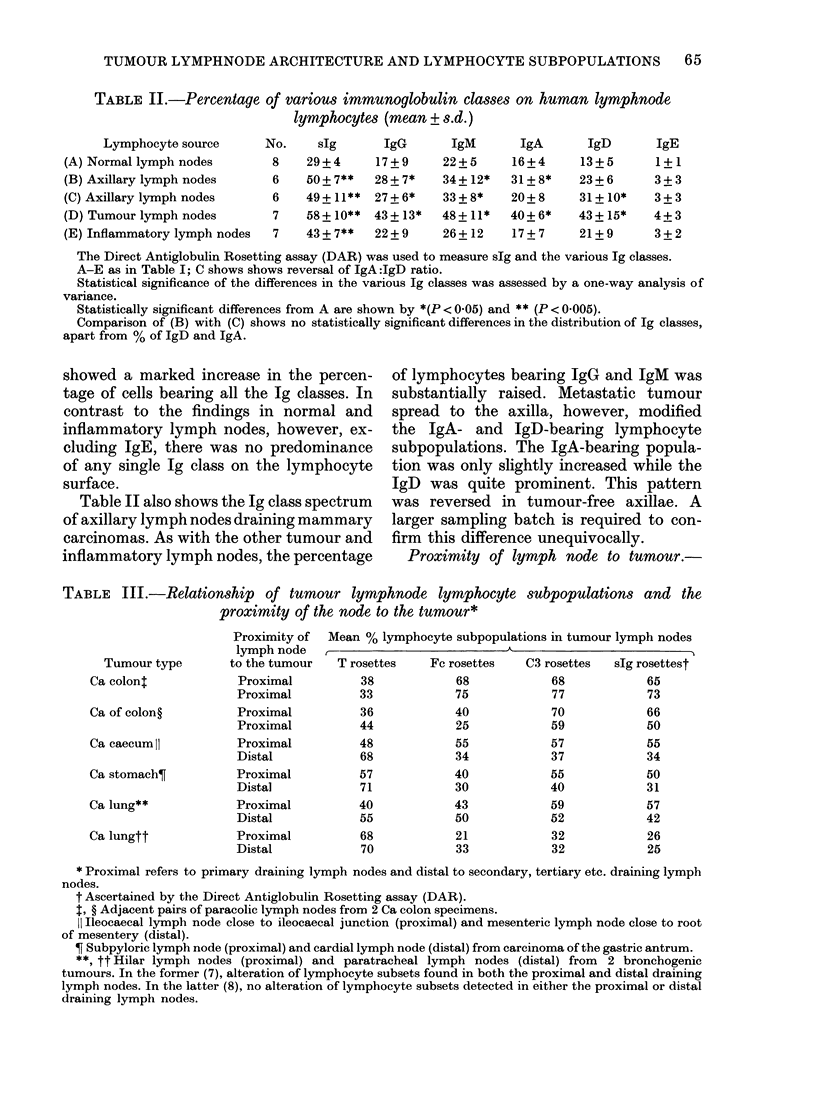

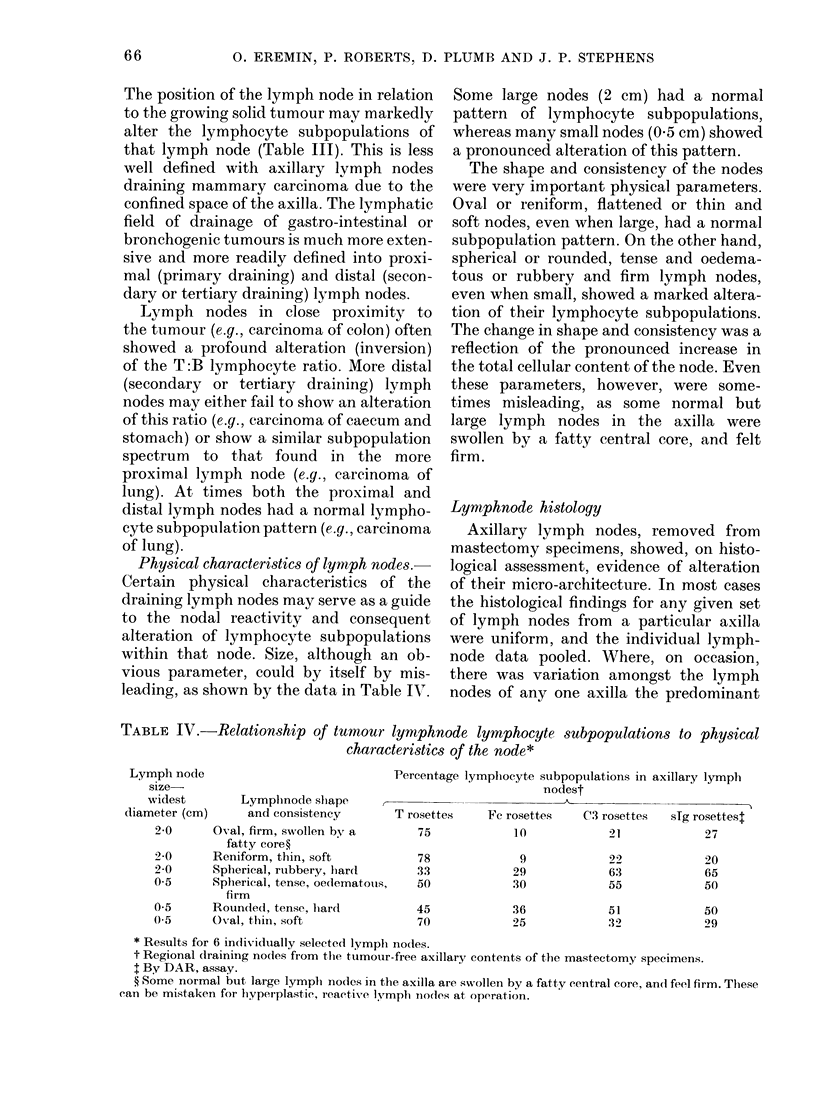

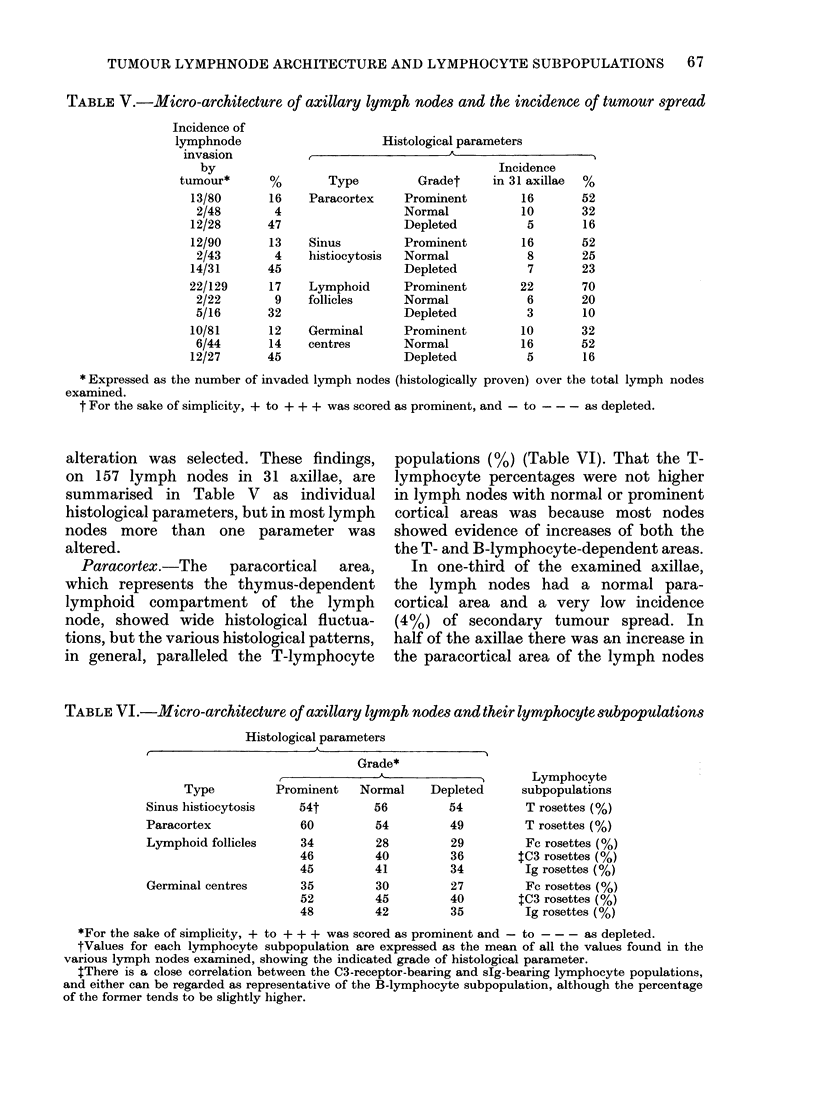

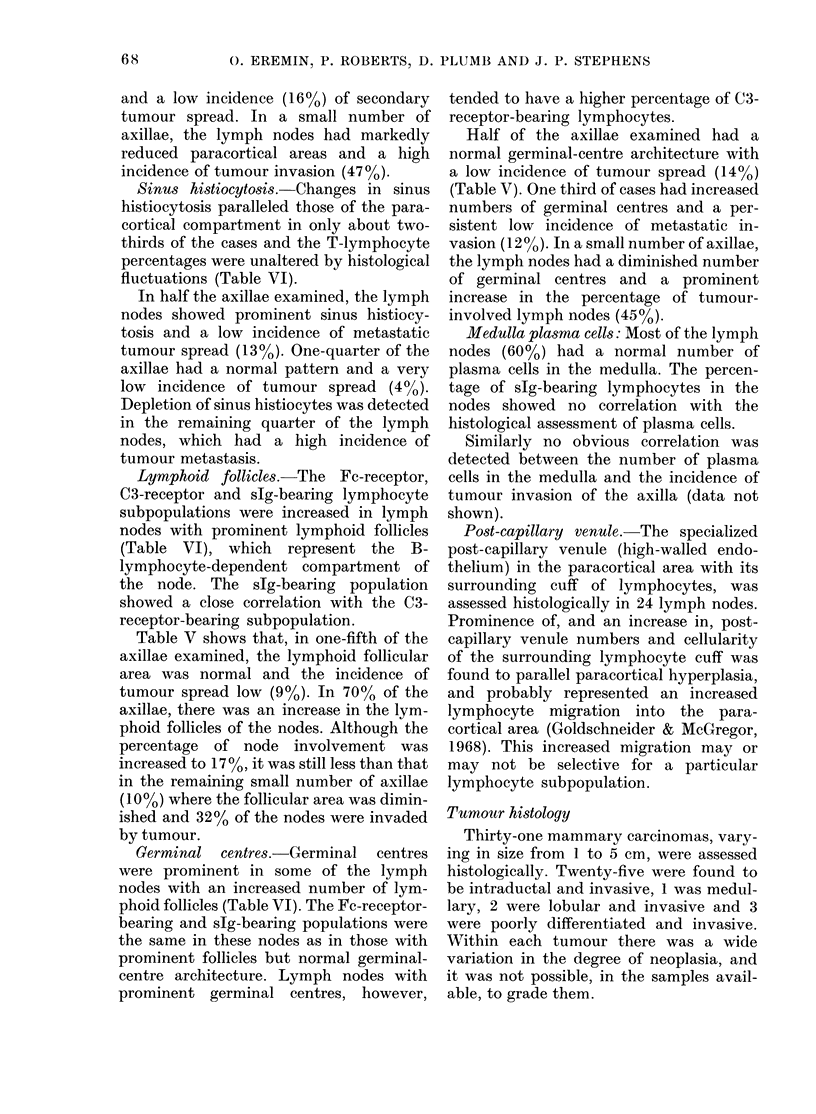

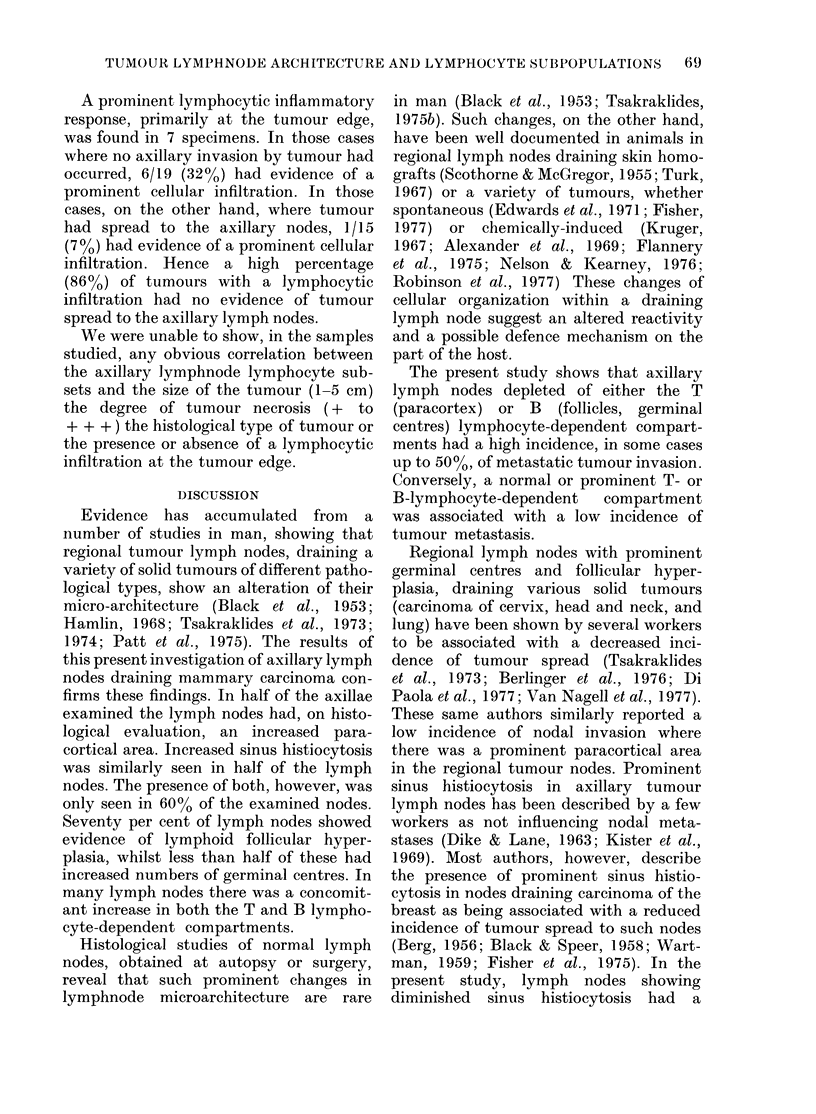

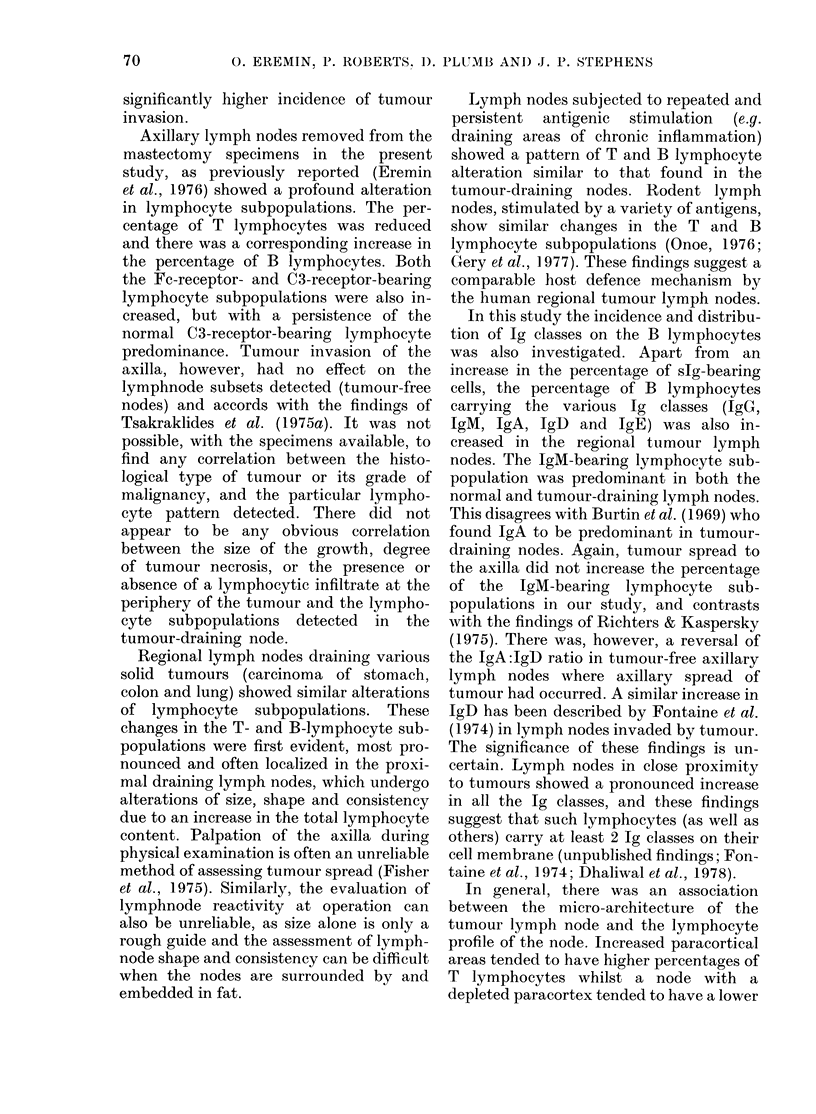

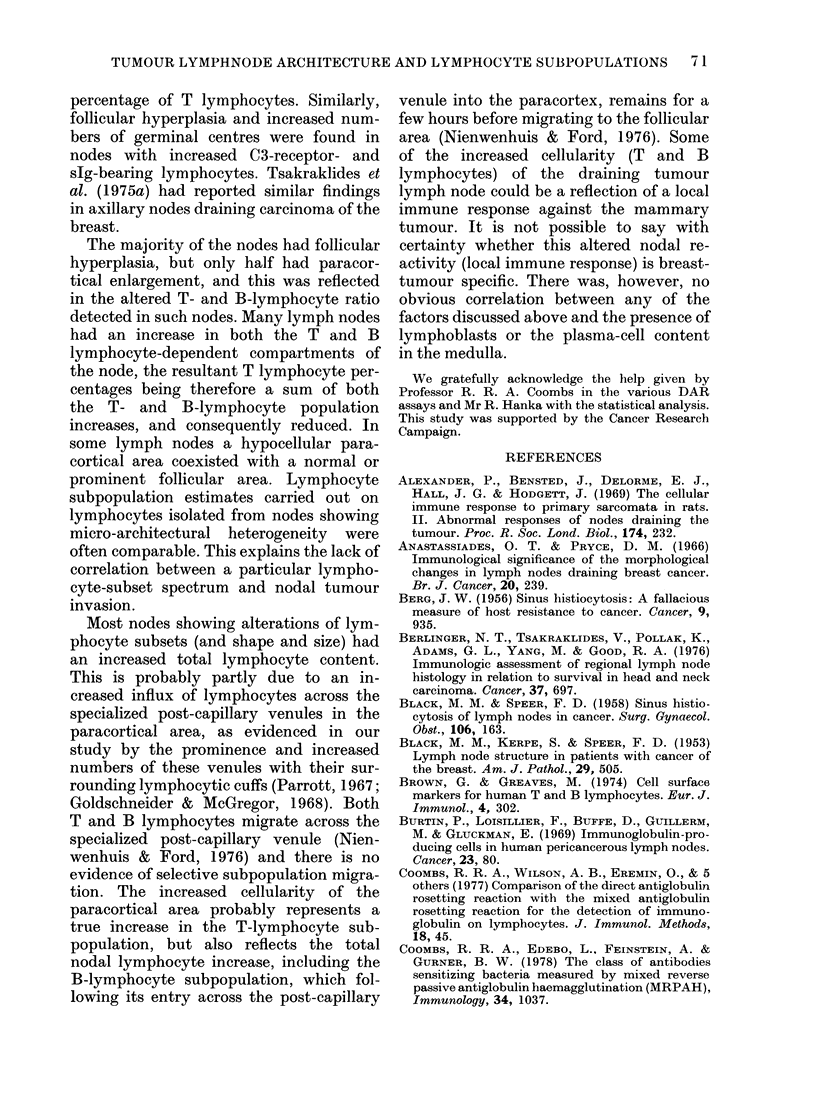

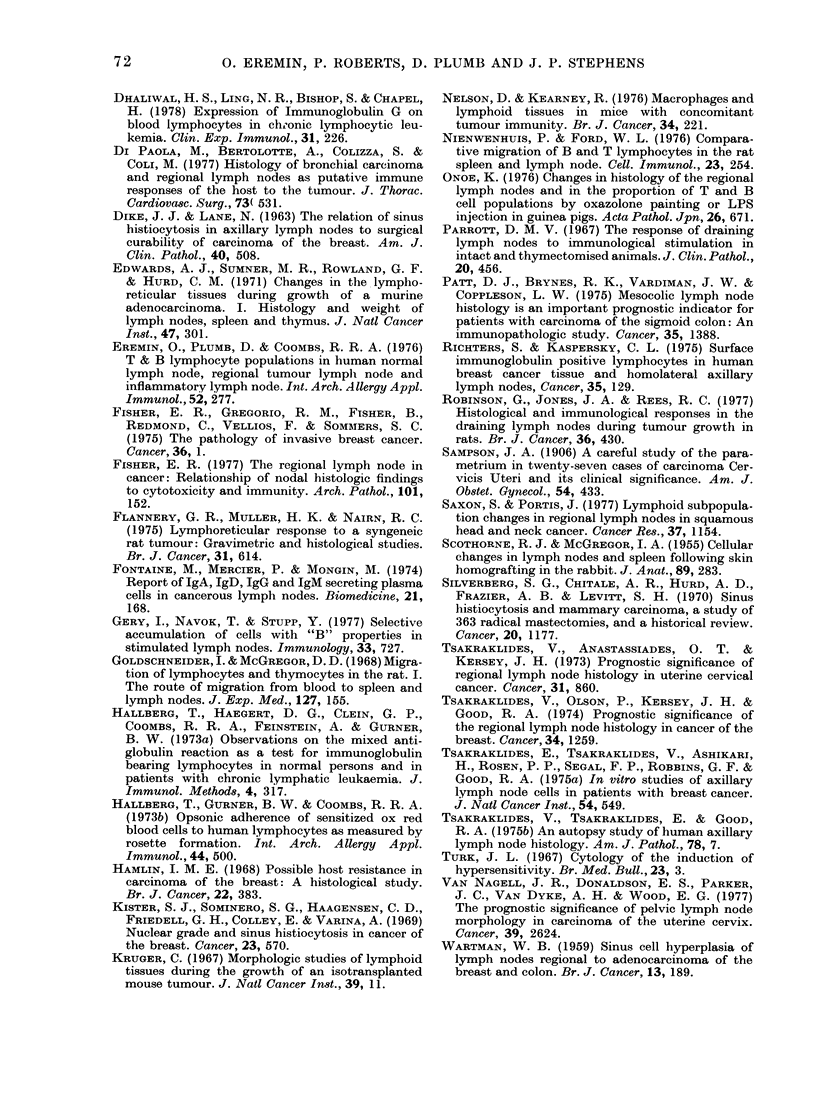


## References

[OCR_01194] Anastassiades O. T., Pryce D. M. (1966). Immunological significance of the morphological changes in lymph nodes draining breast cancer.. Br J Cancer.

[OCR_01200] BERG J. W. (1956). Sinus histiocytosis: a fallacious measure of host resistance to cancer.. Cancer.

[OCR_01217] BLACK M. M., KERPE S., SPEER F. D. (1953). Lymph node structure in patients with cancer of the breast.. Am J Pathol.

[OCR_01212] BLACK M. M., SPEER F. D. (1958). Sinus histiocytosis of lymph nodes in cancer.. Surg Gynecol Obstet.

[OCR_01205] Berlinger N. T., Tsakraklides V., Pollack K., Adams G. L., Yang M., Good R. A. (1976). Immunologic assessment of regional lymph node histology in relation to survival in head and neck carcinoma.. Cancer.

[OCR_01222] Brown G., Greaves M. F. (1974). Cell surface markers for human T and B lymphocytes.. Eur J Immunol.

[OCR_01227] Burtin P., Loisillier F., Buffe D., Guillerm M., Gluckman E. (1969). Immunoglobulin-producing cells in human pericancerous lymph nodes.. Cancer.

[OCR_01241] Coombs R. R., Edebo L., Feinstein A., Gurner B. W. (1978). The class of antibodies sensitizing bacteria measured by mixed reverse passive antiglobulin haemagglutination (MRPAH).. Immunology.

[OCR_01263] DIRE J. J., LANE N. (1963). THE RELATION OF SINUS HISTIOCYTOSIS IN AXILLARY LYMPH NODES TO SURGICAL CURABILITY OF CARCINOMA OF THE BREAST.. Am J Clin Pathol.

[OCR_01250] Dhaliwal H. S., Ling N. R., Bishop S., Chapel H. (1978). Expression of immunoglobin G on blood lymphocytes in chronic lymphocytic leukaemia.. Clin Exp Immunol.

[OCR_01256] Di Paola M., Bertolotti A., Colizza S., Coli M. (1977). Histology of bronchial carcinoma and regional lymph nodes as putative immune response of the host to the tumor.. J Thorac Cardiovasc Surg.

[OCR_01269] Edwards A. J., Sumner M. R., Rowland G. F., Hurd C. M. (1971). Changes in lymphoreticular tissues during growth of a murine adenocarcinoma. I. Histology and weight of lymph nodes, spleen, and thymus.. J Natl Cancer Inst.

[OCR_01277] Eremin O., Plumb D., Coombs R. R. (1976). T and B lymphocyte populations in human normal lymph node, regional tumour lymph node and inflammatory lymph node.. Int Arch Allergy Appl Immunol.

[OCR_01290] Fisher E. R., Fisher B., Saffer E. (1977). The regional lymph node in cancer. Relationship of nodal histologic findings to cytotoxicity and immunity.. Arch Pathol Lab Med.

[OCR_01284] Fisher E. R., Gregorio R. M., Fisher B., Redmond C., Vellios F., Sommers S. C. (1975). The pathology of invasive breast cancer. A syllabus derived from findings of the National Surgical Adjuvant Breast Project (protocol no. 4).. Cancer.

[OCR_01296] Flannery G. R., Muller H. K., Nairn R. C. (1975). Lymphoreticular response to a syngeneic rat tumour: gravimetric and histological studies.. Br J Cancer.

[OCR_01302] Fontaine M., Mercier P., Mongin M. (1974). Report of Ig Ag, Ig D, Ig G and Ig M secreting plasma cells in cancerous lymph nodes.. Biomedicine.

[OCR_01308] Gery I., Navok T., Stupp Y. (1977). Selective accumulation of cells with 'B' properties in stimulated lymph nodes.. Immunology.

[OCR_01313] Goldschneider I., McGregor D. D. (1968). Migration of lymphocytes and thymocytes in the rat. I. The route of migration from blood to spleen and lymph nodes.. J Exp Med.

[OCR_01319] Hallberg T., Haegert D., Clein G. P., Coombs R. R., Feinstein A., Gurner B. W. (1974). Observations on the mixed antiglobulin reaction as a test for immunoglobulin-bearing lymphocytes in normal persons and in patients with chronic lymphatic leukaemia.. J Immunol Methods.

[OCR_01335] Hamlin I. M. (1968). Possible host resistance in carcinoma of the breast: a histological study.. Br J Cancer.

[OCR_01340] Kister S. J., Sommers S. C., Haagenses C. D., Friedell G. H., Cooley E., Varma A. (1969). Nuclear grade and sinus histiocytosis in cancer of the breast.. Cancer.

[OCR_01351] Nelson D. S., Kearney R. (1976). Macrophages and lymphoid tissues in mice with concomitant tumour immunity.. Br J Cancer.

[OCR_01356] Nieuwenhuis P., Ford W. L. (1976). Comparative migration of B- and T-Lymphocytes in the rat spleen and lymph nodes.. Cell Immunol.

[OCR_01360] Onoé K. (1976). Changes in histology of the regional lymph nodes and in the proportions of T and B cell populations by oxazolone painting or LPS injection in guinea pigs.. Acta Pathol Jpn.

[OCR_01371] Patt D. J., Brynes R. K., Vardiman J. W., Coppleson L. W. (1975). Mesocolic lymph node histology is an important prognostic indicator for patients with carcinoma of the sigmoid colon: an immunomorphologic study.. Cancer.

[OCR_01378] Richters A., Kaspersky C. L. (1975). Surface immunoglobulin positive lymphocytes in human breast cancer tissue and homolateral axillary lymph nodes.. Cancer.

[OCR_01401] SCOTHORNE R. J., MCGREGOR I. A. (1955). Cellular changes in lymph nodes and spleen following skin homografting in the rabbit.. J Anat.

[OCR_01396] Saxon A., Portis J. (1977). Lymphoid subpopulation changes in regional lymph nodes in squamous head and neck cancer.. Cancer Res.

[OCR_01406] Silverberg S. G., Chitale A. R., Hind A. D., Frazier A. B., Levitt S. H. (1970). Sinus histiocytosis and mammary carcinoma. Study of 366 radical mastectomies and an historical review.. Cancer.

[OCR_01425] Tsakraklides E., Tsakraklides V., Ashikari H., Rosen P. P., Siegal F. P., Robbins G. F., Good R. A. (1975). In vitro studies of axillary lymph node cells in patients with breast cancer.. J Natl Cancer Inst.

[OCR_01413] Tsakraklides V., Anastassiades O. T., Kersey J. H. (1973). Prognostic significance of regional lymph node histology in uterine cervical cancer.. Cancer.

[OCR_01419] Tsakraklides V., Olson P., Kersey J. H., Good R. A. (1974). Prognostic significance of the regional lymph node histology in cancer of the breast.. Cancer.

[OCR_01432] Tsakraklides V., Tsakraklides E., Good R. A. (1975). An autopsy study of human axillary lymph node histology.. Am J Pathol.

[OCR_01437] Turk J. L. (1967). Cytology of the induction of hypersensitivity.. Br Med Bull.

[OCR_01441] van Nagell J. R., Donaldson E. S., Parker J. C., van Dyke A. H., Wood E. G. (1977). The prognostic significance of pelvic lymph node morphology in carcinoma of the uterine cervix.. Cancer.

